# Association between urinary heavy metal/trace element concentrations and kidney function: a prospective study

**DOI:** 10.1093/ckj/sfae378

**Published:** 2024-11-23

**Authors:** Sisi Xie, Maïwenn Perrais, Déla Golshayan, Gregoire Wuerzner, Julien Vaucher, Aurélien Thomas, Pedro Marques-Vidal

**Affiliations:** Department of Medicine, Internal Medicine, Lausanne University Hospital (CHUV) and University of Lausanne, Lausanne, Switzerland; Faculty Unit of Toxicology, University Centre of Legal Medicine Lausanne-Geneva, Lausanne University Hospital and University of Lausanne, Lausanne, Switzerland; Unit of Forensic Toxicology and Chemistry, CURML, Lausanne and Geneva University Hospitals, Lausanne, Geneva, Switzerland; Transplantation Center, Lausanne University Hospital, Lausanne, Switzerland; Service of Nephrology and Hypertension, Lausanne University Hospital and University of Lausanne, Lausanne, Switzerland; Department of Medicine, Internal Medicine, Lausanne University Hospital (CHUV) and University of Lausanne, Lausanne, Switzerland; Department of Medicine and Specialties, Internal Medicine, Fribourg Hospital and University of Fribourg, Fribourg, Switzerland; Faculty Unit of Toxicology, University Centre of Legal Medicine Lausanne-Geneva, Lausanne University Hospital and University of Lausanne, Lausanne, Switzerland; Unit of Forensic Toxicology and Chemistry, CURML, Lausanne and Geneva University Hospitals, Lausanne, Geneva, Switzerland; Department of Medicine, Internal Medicine, Lausanne University Hospital (CHUV) and University of Lausanne, Lausanne, Switzerland

**Keywords:** chronic kidney disease, epidemiology, glomerular filtration rate, heavy metals, trace elements

## Abstract

**Background:**

Chronic kidney disease (CKD) is an important public health problem. Although cross-sectional studies have identified many heavy metals/trace elements associated with reduced kidney function, prospective studies are needed to determine the pathogenic role of these elements in the development and progression of CKD.

**Methods:**

To explore the association between baseline urinary heavy metal/trace element concentrations and long-term impaired kidney function (IKF)/CKD, as well as the incidence of rapid decline in kidney function in a population-based exploratory prospective study, with mean age 51.9 years at baseline whose urinary trace elements concentrations have been determined by inductively coupled plasma mass spectrometry. IKF was defined by a reduced estimated glomerular filtration rate (eGFR) between 60 and 90 mL/min/1.73 m^2^, and CKD was defined as an eGFR <60 mL/min/1.73 m^2^. Rapid eGFR decline was defined as a decrease ≥3 mL/min/1.73 m^2^/year.

**Results:**

Over a mean follow-up of 12.5 years, 123 participants (2.6%) experienced rapid decline in kidney function, and 1455 (31.7%) developed IKF or CKD. After adjusting for covariates including baseline eGFR, we found that urinary vanadium [hazard ratio (HR) = 1.07, 1.03–1.12], cobalt (HR = 1.69, 1.21–2.37), nickel (HR = 1.19, 1.08–1.3), copper (HR = 1.03, 1.01–1.06), selenium (HR = 1.33, 1.02–1.73), molybdenum (HR = 1.48, 1.2–1.82) and iodine (HR = 1.1, 1.02–1.2) were associated with an increased risk of new incident IKF or CKD cases during the follow-up. Also, urinary copper [odds ratio (OR) = 1.12, 1.04–1.21], silver (OR = 1.83, 1–3.35), molybdenum (OR = 1.02, 1.01–1.04) and cadmium (OR = 1.05, 1.01–1.09) were associated with an increased risk of rapid eGFR decline.

**Conclusion:**

In the general population, several urinary heavy metals/trace elements are associated with a rapid decline in kidney function or new cases of IKF/CKD.

## Graphical Abstract

**Graphical Abstract:**
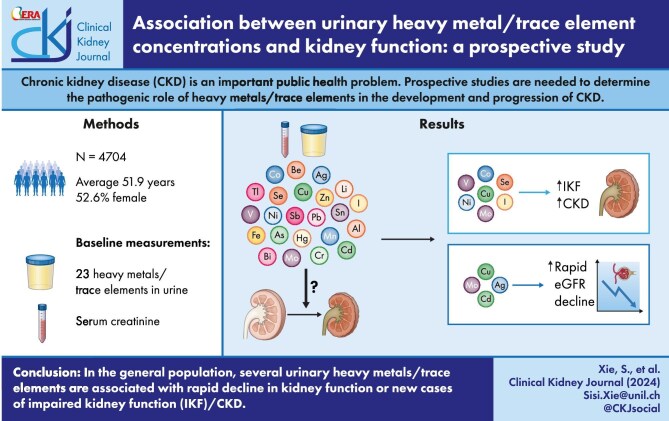

KEY LEARNING POINTS **What was known:**

- With the intensification of global environmental pollution, kidney dysfunction caused by heavy metals is becoming a global public health problem.
- Previous studies have linked heavy metal/trace element exposure to kidney dysfunction. However, the association between kidney function and heavy metals/trace elements remains unclear.

**This study adds:**

- Participants with incident impaired kidney function (IKF) or CKD have higher urinary vanadium, cobalt, nickel, copper, selenium, molybdenum, silver and iodine levels at baseline.
- Urinary vanadium, cobalt, nickel, copper, selenium, molybdenum and iodine were associated with an increased risk of incident IKF or CKD; urinary copper, silver, molybdenum and cadmium were associated with an increased risk of a rapid decline in estimated glomerular filtration rate (eGFR).
- Urinary aluminium, vanadium, manganese, cobalt, copper, selenium, molybdenum, silver, cadmium, tin and iodine levels are significantly and positively associated with annual decline in eGFR.

**Potential impact:**

- This study provides important evidence for early intervention in the general population (e.g. water filtration, annual trace elements screening, etc.) to reduce the risk of CKD.
- This study also provides important evidence for predicting the progression of CKD.

## INTRODUCTION

Chronic kidney disease (CKD) is one of the fastest growing causes of death globally [1]. With the intensification of global environmental pollution, kidney dysfunction caused by heavy metals is becoming a global public health problem. Heavy metals are defined as metals with relatively high density, atomic number or atomic weight [2]. They enter the human body through ingestion, inhalation and skin absorption. Even at low exposure levels, these metals can damage various organs [3], especially the kidney, which is the main target organ for heavy metal metabolism. Chronic exposure to heavy metals or trace elements (environmental or occupational) may lead to elevated blood concentrations with end-organ damages such as tubulointerstitial injury in the kidney with subsequent dysfunction. Conversely, when kidney function is impaired, heavy metal or trace elements excretion disorders may occur. The biological pathways underlying kidney damage differ between the various metal/trace elements with mechanisms such as oxidative stress, lipid peroxidation, mitochondrial dysfunction, impaired DNA repair mechanisms, reduced antioxidant capacity and apoptosis can lead to proximal tubule dysfunction, interstitial fibrosis and tubular atrophy, with subsequently reduced estimated glomerular filtration rate (eGFR) [3]. Existing research shows that lead (Pb), cadmium (Cd), mercury (Hg), arsenic (As), chromium (Cr) and lithium (Li) are common toxic heavy metals that can significantly and irreversibly damage the kidney [4–6]. Identifying which urinary heavy metals/trace elements are associated with increased progression to end-stage kidney disease could provide timely and effective intervention.

Therefore, we conducted an exploratory prospective cohort study to evaluate the association between urinary heavy metals/trace elements and changes in eGFR, and to explore the association between baseline urinary heavy metal/trace element concentrations and long-term impaired kidney function (IKF)/CKD, as well as the incidence of rapid decline in kidney function.

## MATERIALS AND METHODS

### Research design and participants

The CoLaus|PsyCoLaus study is a population-based study initiated in 2003 to investigate the epidemiology and genetic determinants of cardiovascular risk factors [7]. The baseline survey was conducted between 2003 and 2006 and included 6733 participants aged between 35 and 75 years. Subsequently, follow-up surveys were conducted in 2009–12, 2014–17 and 2018–21. Within each survey, participants were invited to attend and to provide urinary samples on-site, after an overnight fasting period. Urine was aliquoted and frozen at –80°C before assessment. Within a nested project focusing on environmental pollutants, the ToxiLaus study, 23 trace elements were measured in urine of 6404 participants.

### Measurement of 23 heavy metals and trace elements in urine

Urine samples (200 µL) were diluted with 1.8 mL of HNO_3_ 1% solution containing 10 ng/mL rhodium and 10 ng/mL indium as internal standards [8, 9]. Samples were analysed using an inductively coupled plasma mass spectrometer (ICP-MS, 7800 Series, Agilent). Using external calibration, quantitative analysis was simultaneously performed on 23 elements: silver (Ag), aluminium (Al), As, beryllium (Be), bismuth (Bi), Cd, cobalt (Co), Cr, copper (Cu), iron (Fe), Hg, iodine (I), Li, manganese (Mn), molybdenum (Mo), nickel (Ni), Pb, antimony (Sb), selenium (Se), tin (Sn), thallium (Tl), vanadium (V) and zinc (Zn). Supplementary data, Table S1 lists the abbreviations, full names and percentile distribution of 23 heavy metals and trace elements included in the study.

Metal values below the limit of detection (LOD) were calculated as the corresponding LOD divided by 2. In this study, the LOD for each heavy metal/trace element was determined and is detailed in Supplementary data, Table S2. The accuracy and precision of the method were routinely assessed using two commercial ClinCheck internal quality controls (http://www.recipe.de/en/index.html). Within the accreditation process, the method is also assessed three times a year with the QMEQAS external quality control organized by the public health expertise and reference centre of Québec (qmeqas, http://www.inspq.qc.ca/ctqenglish/eqas/qmeqas/description).

### Measurement of kidney function

We selected eGFR value and CKD as the primary outcome measures to evaluate participant's kidney function. Serum creatinine levels were measured using the Jaffe method to estimate glomerular filtration rate. eGFR was calculated according to the Chronic Kidney Disease Epidemiology Collaboration 2021 (CKD-EPI 2021) equation [10]. Creatinine/eGFR was measured four times at each survey in the CoLaus study and we checked the value to confirm that the eGFR is still low. Due to study constraints, it was not possible to assess eGFR regularly. Hence, it was impossible to assess whether the low eGFR values were present for at least 3  months, as per clinical definition. IKF, also known as stage 2 CKD, was defined as an eGFR between 60 and 90 mL/min/1.73 m^2^ body surface area, and CKD was defined as an eGFR <60 mL/min/1.73 m^2^ body surface area [11].

Secondary outcomes included annual eGFR decline, defined as (eGFR at baseline – eGFR at follow-up)/follow-up years. A more rapid decline in kidney function is known to be associated with an increased risk of adverse clinical outcomes. We defined rapid decline in kidney function as an annual decline in eGFR ≥3 mL/min/1.73 m^2^ [12–14]. For the rapid decline, the date of the last follow-up was selected for the calculations. We also computed the relative decrease as the ratio between the absolute decrease and the initial values. As albuminuria was unavailable for all surveys, we decided not to use it.

### Covariates

We selected potential confounding factors based on the literature on the relationship between heavy metals and trace elements and kidney function [15, 16]. We selected age (years), sex (male/female), education (low/medium/high), marital status (living alone/living in couple), weekly alcohol consumption (units), smoking (Never/Former/Current), hypertension (yes/no), diabetes (yes/no), body mass index (BMI) (normal/overweight/obese), physical activities (never/once a week/twice a week), triglycerides, uric acid, C-reactive protein, 25-hydroxyvitamin D3 (continuous), baseline eGFR levels (continuous), mean follow-up time (continuous) and urinary osmolarity (mOsm/H_2_O kg). Details of the covariates are shown in Supplementary data.

Participants reported all medicines prescribed or bought over the counter. Medicines were coded according to the Anatomic, Therapeutic and Chemical (ATC) classification of the World Health Organization. Li treatment was defined by codes N05AN* and D11AX04. I-containing drugs were defined by codes H03C*, D08AG* and H03AA*, where * = any code.

### Ethical statement

The institutional Ethics Committee of the University of Lausanne, which afterwards became the Ethics Commission of Canton Vaud (www.cer-vd.ch) approved the baseline CoLaus study (reference 16/03, decisions of 13 January and 10 February 2003). The approval was renewed for the first (reference 33/09, decision of 23 February 2009), the second (reference 26/14, decision of 11 March 2014) and the third (reference PB_2018–00 040, decision of 20 March 2018) follow-ups. The approval for the entire CoLaus|PsyCoLaus study was confirmed in 2021 (reference PB_2018–00 038, 239/09, decision of 21 June 2021). The full decisions of the CER-VD can be obtained from the authors upon request. The study was performed in agreement with the Helsinki declaration and its former amendments and in accordance with the applicable Swiss legislation (LRH 810.30, approved by the Swiss Federal Parliament on 30 September 2011). All participants gave their signed informed consent before entering the study.

### Exclusion criteria

For this study, all participants at baseline were considered as eligible. We then excluded participants with (i) missing baseline trace element data; (ii) missing serum creatinine; (iii) missing covariates (missing at least one of the covariates defined previously); (iv) receiving I and Li therapy at the time of urine testing; and (v) missing laboratory results at follow-up. For the prospective study assessing incidence of IKF, only participants with normal kidney function at baseline were considered. For prospective studies assessing the incidence of CKD, participants with normal kidney function or IKF at baseline were considered. To exclude extreme values from adversely affecting statistical results and to improve the stability and reliability of the model, when analyzing each individual heavy metal/trace element, participants whose urine concentration exceeded the 95% upper limit were also excluded.

### Statistical analysis

Statistical analyses were conducted using Stata v.18 (Stata Corp, College Station, TX, USA). Descriptive results were expressed as number of participants (percentage) for categorical variables and as average ± standard deviation or median (interquartile range) for continuous variables. Between-group comparisons were conducted using chi-square for categorical variables and Student's *t*-test, analysis of variance (ANOVA) or Kruskal–Wallis test for continuous variables. Multivariable comparisons were performed using ANOVA adjusting for the covariates defined previously, the results were expressed as adjusted average ± standard error.

Multiple linear regression and logistic regression analysis were used to evaluate the association between baseline heavy metal/trace element levels and changes in eGFR levels during follow-up. As there are no established thresholds, we ran a sensitivity analysis between the relative change in eGFR and urinary trace elements via Spearman correlation. In addition, to evaluate the effects of baseline concentrations of 23 heavy metals/trace elements on the long-term incidence of new cases of IKF and CKD, we used Cox regression models to estimate hazard ratio (HR) values and 95% confidence intervals (CI) for the association between urinary heavy metals/trace elements and new cases of IKF or CKD during follow-up. Participants whose urine concentrations exceeded the 95% upper limit when analyzing each trace element were excluded. The 25th, 50th (median), 75th and 95th percentiles along with maximum values for all elements are included in Supplementary data, Table S1. We also conducted a sensitivity analysis that included these extreme values, with the results presented in Supplementary data, Table S3.

Multivariable models were adjusted for age, sex, BMI categories (normal, overweight, obese), education (low/medium/high), marital status (alone, in couple), smoking (never, former, current), alcohol consumption (none, 1–13, 14–27 and 28+ per week), hypertension (yes, no), diabetes (yes, no), urinary osmolarity (continuous), physical activity (never, once or twice per week), triglycerides (TG), uric acid, C-reactive protein, 25-hydroxyvitamin D3 (all continuous), eGFR levels (continuous) and mean follow-up time (continuous).

Statistical significance was considered for a two-sided test with *P  *< .05. As this was an exploratory study, we decided not to correct for multiple comparisons.

## RESULTS

### Selection of participants

**Figure 1:**
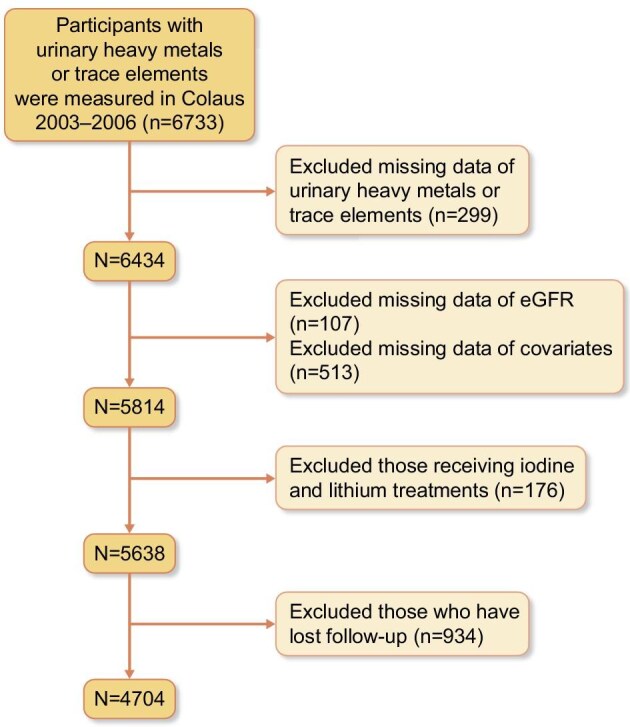
Selection of participants from CoLaus 2003–06. CoLaus study, Lausanne, Switzerland.

A total of 4704 participants were included in this analysis (Fig. 1). The characteristics of included and excluded participants are indicated in Supplementary data, Table S4. We excluded (i) participants with missing baseline trace element data (*n* = 299); (ii) participants with missing GFR (*n* = 107); (iii) participants with missing covariates (*n* = 513); (iv) participants receiving I and Li therapy (*n* = 176); and (v) participants with missing laboratory results at follow-up (*n* = 934). Compared with the included participants, the excluded participants were older, had relatively lower education and drinking levels, and more frequently did not engage in physical activity. Excluded participants were more likely to suffer from obesity, hypertension, diabetes and CKD, and had higher blood levels of TG, uric acid and C-reactive protein. Excluded participants also had lower blood levels of 25-hydroxyvitamin D3 and lower urinary levels of creatinine and osmolality.

### Association between heavy metal/trace element levels and incidence of IKF or CKD

Among the participants selected for the study (mean age 51.9 years, 52.6% female), 51.3% had a normal eGFR, 46.2% presented with IKF and 2.5% with CKD at baseline. The mean eGFR was 89.7 mL/min/1.73m^2^. Mean follow-up time was 12.5 years, ranging from 4.9 to 17.1 years.

During the follow-up period, kidney function was available for 4587 participants, of whom 1455 (31.7%) developed IKF or CKD. Their clinical characteristics at baseline are shown in Table 1. Compared with participants who did not develop IKF or CKD during follow-up, participants who developed IKF or CKD were older, had lower levels of high-density lipoprotein (HDL)-cholesterol, lower levels of urinary creatinine, 25-hydroxyvitamin D3 and uric acid levels, higher eGFR at baseline, higher frequency of prescription drug use, had greater annual decreases in eGFR, and more hypertension and diabetes (Table 1).

**Table 1:** Participant characteristics at baseline, by IKF or CKD events, occurred at follow-up, and whether rapid decline in kidney function occurred.

		Incident IKF or CKD			Rapid kidney function decline		
Variables	Baseline	Yes (*n* = 1455)	No (*n* = 3132)	*P-*value	Yes (*n* = 123)	No (*n* = 4581)	*P-*value
Age, years	51.9 ± 10.4	53.2 ± 10.8	50.8 ± 9.9	**<.001**	57.8 ± 11.3	51.7 ± 10.4	**< .001**
Female sex, *n* (%)	2476 (52.6)	775 (53.3)	1634 (52.2)	.490	53 (43.1)	2423 (52.9)	**.032**
Education level, *n* (%)				.298			**.001**
Low	2508 (53.4)	794 (54.7)	1650 (52.7)		72 (59.0)	2436 (53.2)	
Middle	1158 (24.6)	357 (24.6)	767 (24.5)		39 (32.0)	1119 (24.5)	
High	1033 (22.0)	302 (20.8)	712 (22.8)		11 (9.0)	1022 (22.3)	
Marital status, *n* (%)				.178			.643
Living alone	1515 (32.2)	447 (30.7)	1025 (32.7)		42 (34.1)	1473 (32.2)	
Living in couple	3187 (67.8)	1007 (69.3)	2106 (67.3)		81 (65.9)	3106 (67.8)	
Smoking status, *n* (%)				.927			**.045**
Never	1903 (40.5)	583 (40.1)	1270 (40.5)		37 (30.1)	1866 (40.8)	
Former	1563 (33.2)	480 (33.0)	1035 (33.1)		45 (36.6)	1518 (33.1)	
Current	1236 (26.3)	391 (26.9)	826 (26.4)		41 (33.3)	1195 (26.1)	
Alcohol consumption, *n* (%)				.309			**.033**
None	1262 (26.8)	377 (25.9)	844 (27.0)		43 (35.0)	1219 (26.6)	
1–13/week	2655 (56.5)	846 (58.1)	1749 (55.8)		55 (44.7)	2600 (56.8)	
14–27/week	621 (13.2)	189 (13.0)	419 (13.4)		22 (17.9)	599 (13.1)	
28+/week	166 (3.5)	43 (3.0)	120 (3.8)		3 (2.4)	163 (3.5)	
BMI groups, *n* (%)				.287			**<.001**
Normal	2341 (49.8)	718 (49.4)	1591 (50.8)		36 (29.3)	2305 (50.3)	
Overweight	1716 (36.5)	524 (36.0)	1135 (36.2)		52 (42.3)	1664 (36.3)	
Obese	647 (13.7)	213 (14.6)	406 (13.0)		35 (28.4)	612 (13.4)	
Hypertension, *n* (%)	1574 (33.5)	538 (37.0)	954 (30.5)	**<.001**	72 (58.5)	1502 (32.8)	**<.001**
Diabetes, %	272 (5.8)	106 (7.3)	144 (4.6)	**<.001**	25 (20.3)	247 (5.4)	**<.001**
Lipids, mmol/L							
Total cholesterol	5.6 ± 1.0	5.5 ± 1.0	5.6 ± 1.0	.352	5.5 ± 1.2	5.6 ± 1.0	.701
LDL-C	3.3 ± 0.9	3.3 ± 0.9	3.3 ± 0.9	.759	3.3 ± 1.0	3.3 ± 0.9	.652
HDL-C	1.6 ± 0.4	1.6 ± 0.4	1.7 ± 0.4	**.023**	1.5 ± 0.4	1.7 ± 0.4	**.003**
Triglycerides	1.4 ± 1.1	1.4 ± 1.2	1.3 ± 1.1	.880	1.6 ± 1.3	1.4 ± 1.1	**.005**
Serum creatinine, µmol/L	79.8 ± 17.4	76.2 ± 12.4	79.9 ± 13.7	**<.001**	76.3 ± 15.3	79.9 ± 17.4	**.025**
Urine creatinine, mg/dL	152.8 ± 74.8	146.3 ± 70.3	156.4 ± 76.6	**<.001**	126.4 ± 53.2	153.5 ± 75.2	**<.001**
Uric acid, mmol/L	310.0 ± 84.5	304.1 ± 83.9	309.4 ± 82.4	**.044**	321.5 ± 93.6	309.6 ± 84.3	.126
Urinary mOsm/H_2_O kg	720.7 ± 212.9	713.5 ± 213.3	727.8 ± 213.1	**.047**	697.4 ± 184.3	721.3 ± 213.6	.263
C-reactive protein (mg/L)	1.2 (0.6–2.6)	1.2 (0.6–2.7)	1.1 (0.6–2.5)	.129	2.1 (0.9–4.3)	1.1 (0.6–2.5)	**<.001**
25-OH, vitamin D3 (nmol/L)	51.4 ± 24.1	49.9 ± 23.5	51.9 ± 24.2	**.009**	42.7 ± 25	51.6 ± 24	**<.001**
New Brunswick drugs	1 (0–3)	2 (1–3)	1 (0–3)	**<.001**	2 (1–5)	1 (0–3)	**<.001**
Physical activity, *n* (%)				.608			**.014**
Never	1610 (34.7)	513 (35.7)	1055 (34.2)		57 (47.1)	1553 (34.4)	
Once a week	486 (10.5)	148 (10.3)	327 (10.6)		11 (9.1)	475 (10.5)	
Twice a week	2544 (54.8)	776 (54.0)	1704 (55.2)		53 (43.8)	2491 (55.1)	
GFR, *n* (%)				**<.001**			**.009**
Normal	2415 (51.3)	1166 (80.1)	1249 (39.9)		79 (64.2)	2336 (51.0)	
IKF	2172 (46.2)	289 (19.9)	1883 (60.1)		40 (32.5)	2132 (46.5)	
CKD	117 (2.5)	/	/		4 (3.3)	113 (2.5)	
Baseline eGFR, mL/min/1.73 m^2^	89.7 ± 14.8	93.0 ± 11.8	89.6 ± 14.3	**<.001**	92.7 ± 13.3	89.7 ± 14.8	**.025**
Annual decline in eGFR (mL/min/1.73 m^2^)	0.5 (–0.1 to 1.0)	1.1 (0.6–1.7)	0.3 (10.3–0.7)	**<.001**	3.9 (3.3–4.9)	0.5 (–0.1 to 0.9)	**<.001**

Results are expressed as number of participants (column percentage) for categorical variables and as average ± standard deviation or median and (interquartile range) for continuous variables. Between-group comparisons were performed using chi-square for categorical variables and Student's *t*-test or Kruskal–-Wallis test for continuous variables.

LDL-C, low-density lipoprotein-cholesterol; HDL-C, high-density lipoprotein-cholesterol; ‘/’, no observations.

The associations between baseline urinary heavy metals/trace elements and incidence of IKF or CKD are shown in Table 2. The results of bivariate analysis showed that compared with participants without worsening kidney function, participants who developed IKF or CKD had significantly higher urinary levels of V, Cr, Co, Ni, Cu, Se, Mo, Ag, Cd, Sn, Pb and I (Table 2). In the multivariable analysis (Table 2), the results for V, Co, Ni, Cu, Se, Mo, Ag and I were confirmed, while the associations for Cr, Cd, Sn and Pb were no longer statistically significant. The associations between incident IKF or CKD and urinary heavy metals/trace elements as modeled by Cox regression are shown in Table 3. Increased urinary levels of V, Co, Ni, Cu, Se, Mo and I were associated with a higher risk of developing IKF or CKD.

**Table 2:** Bivariate and multivariable analysis of the associations between urinary heavy metals/trace elements and worsening kidney function.

	Bivariate			Multivariate		
Metals/trace elements	Normal (*n* = 3132)	Worsening (*n* = 1455)	*P-*value	Normal (*n* = 3233)	Worsening (*n* = 1455)	*P-*value
Li (µg/g creatinine)	21.97 ± 17.67	22.19 ± 16.8	.697	21.93 ± 0.33	22.11 ± 0.49	.763
Be (ng/g creatinine)	1.26 ± 0.90	1.30 ± 0.88	.216	1.28 ± 0.02	1.28 ± 0.02	.960
Al (µg/g creatinine)	4.81 ± 3.70	4.98 ± 3.93	.166	4.81 ± 0.07	4.94 ± 0.11	.301
V (ng/g creatinine)	132.03 ± 67.93	137.96 ± 69.86	**.008**	132.47 ± 1.26	139.17 ± 1.86	**.003**
Cr (µg/g creatinine)	0.23 ± 0.16	0.24 ± 0.16	**.041**	0.23 ± 0.00	0.24 ± 0.00	.114
Mn (µg/g creatinine)	0.26 ± 0.17	0.27 ± 0.16	.111	0.26 ± 0.00	0.26 ± 0.00	.483
Co (µg/g creatinine)	0.20 ± 0.15	0.22 ± 0.17	**.003**	0.20 ± 0.00	0.22 ± 0.00	**<.001**
Ni (µg/g creatinine)	1.06 ± 0.60	1.15 ± 0.65	**<.001**	1.07 ± 0.01	1.15 ± 0.02	**<.001**
Cu (µg/g creatinine)	9.03 ± 2.72	9.37 ± 2.85	**<.001**	9.11 ± 0.05	9.39 ± 0.07	**.002**
Zn (µg/g creatinine)	304.38 ± 153.45	304.03 ± 153.71	.944	306.48 ± 2.81	302.86 ± 4.19	.477
As (ug/g creatinine)	25.76 ± 28.25	24.53 ± 26.68	.176	25.87 ± 0.55	24.80 ± 0.80	.275
Se (µg/g creatinine)	42.63 ± 10.91	43.56 ± 11.29	**.010**	42.99 ± 0.21	43.81 ± 0.31	**.029**
Mo (µg/g creatinine)	29.71 ± 14.02	31.63 ± 13.92	**<.001**	29.77 ± 0.26	31.89 ± 0.39	**<.001**
Ag (ng/g creatinine)	29.66 ± 18.56	31.68 ± 18.61	**.001**	30.03 ± 0.29	31.24 ± 0.43	**.023**
Cd (µg/g creatinine)	0.47 ± 0.28	0.49 ± 0.30	**.006**	0.47 ± 0.00	0.48 ± 0.01	.881
Sn (µg/g creatinine)	0.63 ± 0.45	0.66 ± 0.46	**.048**	0.64 ± 0.01	0.66 ± 0.01	.215
Sb (ng/g creatinine)	50.33 ± 33.19	50.26 ± 32.44	.944	50.92 ± 0.63	50.31 ± 0.93	.589
Hg (µg/g creatinine)	1.15 ± 0.77	1.16 ± 0.76	.803	1.17 ± 0.01	1.17 ± 0.02	.979
Pb (µg/g creatinine)	1.38 ± 0.78	1.44 ± 0.77	**.014**	1.41 ± 0.01	1.43 ± 0.02	.477
Tl (µg/g creatinine)	0.19 ± 0.08	0.19 ± 0.08	.282	0.19 ± 0.00	0.19 ± 0.00	.687
Bi (ng/g creatinine)	5.77 ± 4.86	6.04 ± 4.73	.095	5.85 ± 0.08	5.90 ± 0.12	.728
Fe (µg/g creatinine)	5.24 ± 3.40	5.37 ± 3.43	.238	5.24 ± 0.06	5.34 ± 0.09	.378
I (µg/g creatinine)	93.87 ± 34.86	98.68 ± 35.87	**<.001**	94.68 ± 0.66	98.26 ± 0.99	**.003**

Results are expressed as average ± standard deviation for bivariate analysis and as multivariable-adjusted average ± standard error. Between group comparisons were done using *t*-test (bivariate) or ANOVA adjusted for age, sex, BMI categories (normal, overweight, obese), education (low/medium/high), marital status (alone, in couple), smoking (never, former, current), alcohol consumption (none, 1–13, 14–27 and 28+ per week), hypertension (yes, no), diabetes (yes, no), urinary osmolarity (continuous), physical activity (never, once or twice per week), triglycerides, uric acid , C-reactive protein, 25-hydroxyvitamin D3 (all continuous), eGFR levels (continuous) and mean follow-up time (continuous).

**Table 3:** Risk of rapid decline in kidney function and new IKF or CKD events.

	Incident IKF or CKD		Rapid kidney function decline	
Abbreviations	HR (95% CI)	*P-*value	OR (95% CI)	*P-*value
Li^a^	0.99 (0.84–1.16)	.889	1.05 (0.58–1.90)	.865
Be	1.00 (0.94–1.07)	.912	1.01 (0.78–1.30)	.944
Al	1.01 (0.99–1.02)	.354	1.02 (0.96–1.08)	.521
V^a^	1.07 (1.03–1.12)	**.001**	1.06 (0.91–1.24)	.435
Cr^a^	1.02 (1.00–1.03)	.075	1.06 (1.00–1.12)	.071
Mn^a^	1.01 (0.99–1.03)	.344	1.06 (0.98–1.14)	.125
Co	1.69 (1.21–2.37)	**.002**	1.22 (0.28–5.34)	.793
Ni	1.19 (1.08–1.30)	**<.001**	1.16 (0.81–1.64)	.419
Cu	1.03 (1.01–1.06)	**.004**	1.12 (1.04–1.21)	**.003**
Zn^a^	0.99 (0.97–1.01)	.253	1.00 (0.93–1.07)	.991
As^a^	0.92 (0.83–1.02)	.116	0.98 (0.65–1.48)	.923
Se^a^	1.33 (1.02–1.73)	**.036**	1.02 (1.00–1.04)	.080
Mo^a^	1.48 (1.20–1.82)	**<.001**	1.02 (1.01–1.04)	**.008**
Ag^a^	1.17 (0.98–1.40)	.079	1.83 (1.00–3.35)	**.049**
Cd^a^	1.00 (0.99–1.01)	.910	1.05 (1.01–1.09)	**.012**
Sn	1.07 (0.95–1.22)	.267	1.35 (0.88–2.08)	.171
Sb	0.73 (0.12–4.44)	.737	0.22 (0.00–133.92)	.646
Hg	1.03 (0.95–1.11)	.505	0.90 (0.65–1.25)	.534
Pb	1.04 (0.96–1.13)	.295	1.08 (0.82–1.43)	.566
Tl^a^	1.02 (0.98–1.06)	.385	1.04 (0.90–1.20)	.596
Bi	1.00(0.99–1.01)	.894	1.01 (0.96–1.06)	.793
Fe	1.01 (0.99–1.02)	.514	1.05 (0.99–1.11)	.079
I^a^	1.10 (1.02–1.20)	**.014**	1.24 (0.92–1.66)	.160

The multivariable associations were assessed using cox regression and logistic regression, results are expressed as HR and OR and corresponding *P*-value.

a HR and OR for a 50-unit increase.

Regressions were adjusted for age, sex, BMI categories (normal, overweight, obese), education (low/medium/high), marital status (alone, in couple), smoking (never, former, current), alcohol consumption (none, 1–13, 14–27 and 28+ per week), hypertension (yes, no), diabetes (yes, no), urinary osmolarity (continuous), physical activity (never, once or twice per week), triglycerides, uric acid , C-reactive protein, 25-hydroxyvitamin D3 (all continuous), eGFR levels (continuous) and mean follow-up time (continuous).

### Association of heavy metal/trace element levels with rapid decline in eGFR

During the follow-up period, the average annual decrease in eGFR was 0.52 mL/min/1.73 m^2^. Overall, 123 (2.6%) participants presented with rapid kidney function decline. Compared with participants without rapid kidney function decline, participants with rapid kidney function decline were older, more frequently male, currently smoking and did not engage in physical activity and consume alcohol, were less likely to have higher education, more likely to suffer from obesity, hypertension and diabetes, a higher frequency of prescription drug use. They also had lower HDL-cholesterol, urinary and serum creatinine, and 25-hydroxyvitamin D3 levels, and higher triglycerides, urinary albumin and C-reactive protein levels. Participants with rapid kidney function decline also had higher eGFR at baseline.

The multivariable analysis of the associations between heavy metals/trace elements and rapid decline in eGFR is shown in Table 3. Cu, Mo, Ag and Cd were associated with an increased risk of rapid eGFR decline (Table 3). To test the robustness of the results, we performed sensitivity analyses to examine the associations of baseline heavy metal/trace element concentrations with the risk of rapid eGFR decline at the first follow-up, and the results confirmed the associations of V, Mo, Ag, and I with rapid eGFR decline (Supplementary data, Table S5).

### Association between heavy metal/trace element levels and annual eGFR changes

The associations between heavy metals/trace elements and annual eGFR changes are shown in Table 4. The results of bivariate analysis showed that the urinary levels of Be, Al, V, Cr, Mn, Cu, Zn, Mo, Ag, Cd, Sn, Pb, Hg, Bi, Fe and I were significantly and positively associated with the annual decline in eGFR, while Hg was negatively associated (Table 4). In the multivariable analysis, the associations were confirmed for Al, V, Mn, Co, Cu, Se, Mo, Ag, Cd, Sn and I (Table 4). In addition, to examine the robustness of the results, we performed sensitivity analyses using relative changes in eGFR. Except for Se, the results of the sensitivity analyses were consistent with the original findings (Supplementary data, Table S6).

**Table 4:** Association between heavy metal/trace element levels and annual eGFR changes.

Metals/trace	Bivariate		Multivariate	
elements	Beta	*P-*value	Beta	*P-*value
Li	0.013	.377	0.020	.200
Be	0.032	**.031**	0.024	.163
Al	0.064	**<.001**	0.054	**.001**
V	0.068	**<.001**	0.065	**<.001**
Cr	0.039	**.009**	0.020	.209
Mn	0.058	**<.001**	0.069	**.001**
Co	0.024	.108	0.044	**.008**
Ni	0.023	.119	0.022	.189
Cu	0.112	**<.001**	0.105	**<.001**
Zn	0.051	**.001**	0.009	.591
As	–0.025	.090	–0.010	.509
Se	0.024	.115	0.036	**.026**
Mo	0.068	**<.001**	0.094	**<.001**
Ag	0.082	**<.001**	0.104	**<.001**
Cd	0.090	**<.001**	0.051	**.006**
Sn	0.042	**.005**	0.041	**.010**
Sb	0.022	.146	–0.003	.873
Hg	-0.031	**.039**	–0.010	.534
Pb	0.043	**.004**	0.016	.342
Tl	0.004	.803	0.016	.321
Bi	0.036	**.017**	0.023	.217
Fe	0.057	**<.001**	0.029	.076
I	0.068	**<.001**	0.061	**<.001**

The multivariable associations were assessed using linear regression, results are expressed as beta for the metals/trace element and corresponding *P*-value.

Regressions were adjusted for age, sex, BMI categories (normal, overweight, obese), education (low/medium/high), marital status (alone, in couple), smoking (never, former, current), alcohol consumption (none, 1–13, 14–27 and 28+ per week), hypertension (yes, no), diabetes (yes, no), urinary osmolarity (continuous), physical activity (never, once or twice per week), triglycerides, uric acid, C-reactive protein, 25-hydroxyvitamin D3 (all continuous), eGFR levels (continuous) and mean follow-up time (continuous).

## DISCUSSION

This study shows that in the general population, urinary Al, V, Mn, Co, Ni, Cu, Se, Mo, Ag, Cd, Sn and I were associated with an increased risk of long-term IKF or CKD development and decline of eGFR. These findings suggest that heavy metal/trace element levels may be an independent predictor of kidney function as assessed by eGFR.

Oxidative stress, lipid peroxidation, mitochondrial dysfunction, impaired DNA repair, decreased antioxidant capacity and cell apoptosis are the main mechanisms of kidney toxicity caused by heavy metals or trace elements [17]. However, the mechanisms of kidney damage caused by different heavy metals or trace elements are not the same. The metals that have been studied more extensively regarding kidney toxicity include Hg, Cd, Pb and As. Hg is mainly reabsorbed in the proximal tubules. Acute Hg exposure can cause mitochondrial and DNA damage, leading to acute tubular epithelial cell necrosis [18, 19]. In addition, Hg also damages the glomeruli by inducing the production of antinuclear autoantibodies, with immune complex deposition in the kidneys [20]. Cd mainly causes irreversible damage to kidney function through oxidative stress, inhibition of DNA damage repair and cell apoptosis [21–23]. Chronic Pb exposure can lead to albuminuria and decreased eGFR [24]. The underlying mechanisms may be related to proximal tubular cell damage caused by oxidative stress and cell apoptosis, glomerular dysfunction and interstitial fibrosis [25]. Acute As poisoning can lead to tubular necrosis and tubulointerstitial nephritis, while chronic exposure can induce oxidative stress with cell damage and death, leading to kidney dysfunction with some reported severe cases of nephrocalcinosis and kidney papillary necrosis [26].

This study was a prospective study focusing on the general population. Consistent with previous studies, our prospective study confirmed that urinary Al, V, Mn, Co, Ni, Cu, Se, Mo, Ag, Cd, Sn and I levels are significantly and positively associated with the risk of IKF or CKD, and the decline of eGFR. A longitudinal study of Chinese adults by Liu *et al*. showed that plasma levels of metals such as Al, As, barium (Ba), Pb, Mo, rubidium (Rb), strontium (Sr), V and Zn were significantly associated with decreased kidney function [27]. In addition, a prospective study of CKD patients by Tsai *et al*. in Taiwan showed that CKD patients who were long-term exposed to As, Cd, Cr, Hg, Cu, Pb, Ni and Zn in the soil were likely to rapidly progress to end-stage kidney disease [28]. Another population-based case–control study by Hu *et al*. in China also found that Pb, Cd, Co and Mn concentrations were significantly associated with CKD risk [29]. In addition to the above-mentioned prospective studies or case–control studies, a population-based cross-sectional study by Yang *et al*. in China showed that plasma As, Mo and urinary Cu are risk factors for abnormal kidney function [16]. Another cross-sectional study by Huang *et al*. showed that Cu, Fe and Zn were positively associated with an increased risk of CKD [30]. In addition, no significant association was found between urinary V and eGFR, but higher V-containing metal mixtures were shown to be associated with reduced annual changes in eGFR [31, 32].

Regarding the research on heavy metal elements in urine, Yu *et al*. [33] measured the association between urinary elements such as As, Cd, Cu, Se and Zn, and eGFR status. They found an association between urinary As, Cd, Cu, Se and Zn and CKD risk, while urinary As, Cu, Se and Zn were associated with IKF risk. Similarly, Sanders *et al*. [4] measured urinary Pb, Cd, Hg and As in US adolescents and showed that As, Pb, Hg, Cd and their combinations may affect kidney parameters.

Although our findings are consistent with some previous studies, the results remain controversial. Conversely, and contrary from previous studies, we found no evidence that As was related to the risk of IKF or CKD, or the decline in annual eGFR. In Switzerland, since As levels are very low in adults, it may not be surprising that we did not find an association between As and measures of kidney function [34]. Regarding Pb and Hg, we found an association only in the bivariate analysis. Regarding I: the association might be due to salt consumption, as salt is the primary source of iodine in Switzerland [35]. Although we adjusted for hypertension, some residual confounding might exist, as no information regarding dietary intake was available.

Moreover, these differences may be attributed to the fact that firstly, most of the results of the previous studies are based on cross-sectional studies [4, 16, 30–33] or case–control studies [29], and due to their inherent limitations, the causal relationship between heavy metals/trace elements and kidney function cannot be well determined. Secondly, the participants in some studies were CKD patients [28], and our study is population-based. Finally, the few prospective studies mentioned above are based mainly on blood samples, and our study is based on urine samples. There is a complex relationship between blood heavy metal/trace element concentrations and urine heavy metal/trace element concentrations, which is affected by multiple factors including exposure route, exposure time, element metabolism, and storage and excretion mechanisms, rather than a simple linear relationship. In future studies, combined analysis with serum values can provide a more comprehensive understanding of the severity of heavy metal and trace element exposure in the body.

An interesting finding of our study is that participants with relatively low eGFR at inclusion were associated with slower annual eGFR decline compared with participants with higher baseline eGFR. This may be related to a hyperfiltration state caused by metabolic diseases such as obesity, hypertension or diabetes, which makes participants more susceptible to kidney disease [36, 37]. Notably, a recent cross-sectional study by Mouti et al. reported significant associations of Cd, Zn, Hg, and Mo with BMI, metabolic syndrome, and smoking status [38]. These findings complement our results by providing insights into potential shared pathways through which these trace elements may impact health.

This study has several strengths. First, our study was based on a large prospective cohort study in Switzerland in which all procedures were highly standardized, participants were selected from the general population and with a long follow-up, rather than from a hospital population as in other observational studies. To our knowledge, this is the first large population-based prospective study reported in Europe measuring the association of up to 23 urinary heavy metals/trace elements with kidney function, providing some evidence of the nephrotoxicity of a subset of these heavy metals/trace elements. Furthermore, this study provides a new perspective on risk assessment and management of metal exposure.

Some limitations of this study are worth mentioning. First, this study lacked direct GFR measurement data and instead used the CKD-EPI equation based on serum creatinine to estimate GFR. However, eGFR based on serum creatinine remains the reference point for kidney function assessment in almost all epidemiological studies, so this is only a minor limitation. Another limitation is the intermittent observation of patients, rather than continuous monitoring of kidney function that would allow a more precise diagnosis of CKD. Still, this would prove unpractical in an epidemiological setting given the large sample size. Second, This prospective study can only generate hypotheses and not demonstrate causality, since the study was exploratory, multiplicity was not corrected and high collinearity between metals/trace elements would complicate the interpretation of multivariate results. In addition, as with any cohort, residual confounding exists. Third, in this study, 1923 participants were excluded, the participants who were excluded from this study were older and had lower eGFR than the included participants. Therefore, there may be selection bias, and the results may not be applicable to the entire population. Fourth, we measured urinary heavy metal/trace element concentrations only once at baseline, lacking knowledge on evolution of trace element concentrations during the follow-up years.

## CONCLUSION

This study found significant associations between several urinary heavy metals/trace elements and decreased kidney function. Further blood studies are necessary to complete our findings and elucidate possible mechanisms.

## Supplementary Material

sfae378_Supplemental_File

## Data Availability

The data of CoLaus|PsyCoLaus study used in this article cannot be fully shared as they contain potentially sensitive personal information on participants. According to the Ethics Committee for Research of the Canton of Vaud, sharing these data would be a violation of the Swiss legislation with respect to privacy protection. However, coded individual-level data that do not allow researchers to identify participants are available upon request to researchers who meet the criteria for data sharing of the CoLaus|PsyCoLaus Datacenter (CHUV, Lausanne, Switzerland). Any researcher affiliated to a public or private research institution who complies with the CoLaus|PsyCoLaus standards can submit a research application to research.colaus@chuv.ch or research.psycolaus@chuv.ch. Proposals requiring baseline data only will be evaluated by the baseline (local) Scientific Committee (SC) of the CoLaus and PsyCoLaus studies. Proposals requiring follow-up data will be evaluated by the follow-up (multicentric) SC of the CoLaus|PsyCoLaus cohort study. Detailed instructions for gaining access to the CoLaus|PsyCoLaus data used in this study are available at www.colaus-psycolaus.ch/professionals/how-to-collaborate/.
