# Supplementary material for: Association between urinary heavy metal/trace element concentrations and kidney function: a prospective study
Source: Clin Kidney J. 2024 Nov 23;18(2):sfae378. doi: 10.1093/ckj/sfae378 (PMC11822291; doi:10.1093/ckj/sfae378)
Supplement: sfae378_Supplemental_File [file sfae378_supplemental_file.docx]

**Supplementary Table 1**: Abbreviations, full names and percentile distribution of the 23 heavy metals/trace elements.

| **Abbreviations** | **Full names** | **25^th^ percentile** | **50^th^ percentile** | **75^th^ percentile** | **95^th^ percentile** | **Maximum** |
| --- | --- | --- | --- | --- | --- | --- |
| Li (ug/g creatinine) | Lithium | 11.32 | 17.04 | 28.85 | 101.86 | 15,351.00 |
| Be (ng/g creatinine) | Beryllium | 0.65 | 1.00 | 1.82 | 4.55 | 558.87 |
| Al (ug/g creatinine) | Aluminum | 2.30 | 3.95 | 6.95 | 19.4 | 15,475.14 |
| V (ng/g creatinine) | Vanadium | 86.25 | 120.76 | 172.13 | 359.90 | 164,129.30 |
| Cr (ug/g creatinine) | Chromium | 0.12 | 0.19 | 0.33 | 0.87 | 601.53 |
| Mn (ug/g creatinine) | Manganese | 0.16 | 0.22 | 0.34 | 0.92 | 134.51 |
| Co (ug/g creatinine) | Cobalt | 0.11 | 0.16 | 0.27 | 0.97 | 99.45 |
| Ni (ug/g creatinine) | Nickel | 0.65 | 0.98 | 1.49 | 3.18 | 694.45 |
| Cu (ug/g creatinine) | Copper | 7.29 | 8.86 | 11.03 | 18.17 | 7,807.28 |
| Zn (ug/g creatinine) | Zinc | 192.1 | 290.53 | 423.76 | 704.97 | 387,414.40 |
| As (ug/g creatinine) | Arsenic | 7.56 | 15.42 | 36.41 | 145.63 | 10,400.83 |
| Se (ug/g creatinine) | Selenium | 35.28 | 42.44 | 51.06 | 72.83 | 52,986.50 |
| Mo (ug/g creatinine) | Molybdenum | 20.03 | 29.82 | 41.39 | 66.57 | 49,920.45 |
| Ag (ng/g creatinine) | Silver | 18.10 | 25.47 | 39.66 | 100.37 | 17,126.81 |
| Cd (ug/g creatinine) | Cadmium | 0.26 | 0.41 | 0.67 | 1.34 | 242.46 |
| Sn (ug/g creatinine) | Tin | 0.33 | 0.53 | 0.88 | 2.45 | 531.33 |
| Sb (ng/g creatinine) | Antimony | 27.60 | 42.79 | 68.25 | 175.31 | 37,188.98 |
| Hg (ug/g creatinine) | Mercury | 0.57 | 1.01 | 1.72 | 3.56 | 898.84 |
| Pb (ug/g creatinine) | Lead | 0.85 | 1.26 | 1.90 | 3.95 | 1,325.68 |
| TI (ug/g creatinine) | Thallium | 0.13 | 0.18 | 0.24 | 0.44 | 144.26 |
| Bi (ng/g creatinine) | Bismuth | 3.23 | 4.47 | 7.10 | 36.48 | 17,342.47 |
| Fe (ug/g creatinine) | Iron | 3.08 | 4.43 | 7.05 | 18.90 | 6,294.78 |
| I (ug/g creatinine) | Iodine | 70.84 | 91.36 | 120.99 | 197.91 | 127,664.20 |

**Supplementary Table 2 Concentration, number and percentage of LOD.**

| **Abbreviations** | **LOD (ug/L)** | **Number of observations below LOD (n and %)** | |
| --- | --- | --- | --- |
| Li | 0.437 | 0 | 0 |
| Be | 0.002 | 3,421 | 72.73 |
| Al | 1.223 | 226 | 5.65 |
| V | 0.024 | 27 | 0.57 |
| Cr | 0.100 | 474 | 10.08 |
| Mn | 0.568 | 4316 | 91.75 |
| Co | 0.023 | 17 | 0.36 |
| Ni | 0.044 | 10 | 0.21 |
| Cu | 2.712 | 71 | 1.51 |
| Zn | 8.596 | 0 | 0 |
| As | 0.117 | 0 | 0 |
| Se | 1.578 | 0 | 0 |
| Mo | 0.054 | 0 | 0 |
| Ag | 0.050 | 4040 | 85.88 |
| Cd | 0.042 | 10 | 0.21 |
| Sn | 0.152 | 180 | 3.83 |
| Sb | 0.044 | 1739 | 36.97 |
| Hg | 0.094 | 24 | 0.51 |
| Pb | 0.226 | 80 | 1.7 |
| Tl | 0.003 | 0 | 0 |
| Bi | 0.100 | 4552 | 96.77 |
| Fe | 0.080 | 6 | 0.13 |
| I | 2.497 | 0 | 0 |

**Supplementary Table 3** Risk of rapid decline in kidney function and new IKF or CKD events contains extreme values.

| **Abbreviations** | **Q1** | **Q2**  **HR (95%CI)** | **Q3**  **HR (95%CI)** | **Q4**  **HR (95%CI)** | ***P* for trend** |
| --- | --- | --- | --- | --- | --- |
| Li | reference | 1.13 (0.96 - 1.32) | 1.05 (0.89 - 1.24) | 1.14 (0.97 - 1.34) | 0.231 |
| Be | reference | 0.99 (0.84 - 1.17) | 1.16 (0.98 - 1.36) | 1.08 (0.91 - 1.29) | 0.165 |
| Al | reference | 0.95 (0.81 - 1.11) | 1.04 (0.89 - 1.21) | 0.99 (0.84 - 1.16) | 0.813 |
| V | reference | 0.93 (0.79 - 1.09) | 1.17 (1.00 - 1.37) | 1.20 (1.02 - 1.41) | **0.003** |
| Cr | reference | 1.14 (0.97 - 1.34) | 1.23 (1.05 - 1.44) | 1.22 (1.04 - 1.43) | **0.011** |
| Mn | reference | 1.00 (0.85 - 1.18) | 1.22 (1.03 - 1.45) | 1.24 (1.01 - 1.52) | **0.011** |
| Co | reference | 1.15 (0.98 - 1.36) | 1.26 (1.06 - 1.50) | 1.37 (1.16 - 1.63) | **<0.001** |
| Ni | reference | 1.03 (0.87 - 1.21) | 1.10 (0.94 - 1.30) | 1.21 (1.03 - 1.43) | **0.016** |
| Cu | reference | 0.94 (0.79 - 1.11) | 1.02 (0.86 - 1.21) | 1.23 (1.04 - 1.46) | **0.009** |
| Zn | reference | 0.91 (0.78 - 1.07) | 0.91 (0.78 - 1.07) | 0.98 (0.84 - 1.16) | 0.852 |
| As | reference | 0.99 (0.85 - 1.16) | 1.03 (0.88 - 1.20) | 0.93 (0.79 - 1.09) | 0.469 |
| Se | reference | 1.05 (0.90 - 1.24) | 1.10 (0.94 - 1.30) | 1.15 (0.98 - 1.35) | 0.076 |
| Mo | reference | 1.23 (1.04 - 1.46) | 1.36 (1.15 - 1.60) | 1.47 (1.24 - 1.74) | **<0.001** |
| Ag | reference | 1.13 (0.96 - 1.33) | 1.19 (1.00 - 1.41) | 1.41 (1.16 - 1.70) | **<0.001** |
| Cd | reference | 1.01 (0.85 - 1.19) | 0.96 (0.81 - 1.15) | 1.00 (0.82 - 1.21) | 0.859 |
| Sn | reference | 1.03 (0.88 - 1.21) | 1.04 (0.88 - 1.22) | 1.04 (0.88 - 1.23) | 0.632 |
| Sb | reference | 1.14 (0.97 - 1.33) | 1.09 (0.93 - 1.28) | 1.03 (0.87 - 1.22) | 0.845 |
| Hg | reference | 1.01 (0.86 - 1.19) | 1.08 (0.92 - 1.27) | 1.07 (0.91 - 1.26) | 0.313 |
| Pb | reference | 0.99 (0.84 - 1.17) | 1.19 (1.01 - 1.40) | 1.16 (0.97 - 1.38) | **0.027** |
| TI | reference | 0.93 (0.79 - 1.09) | 1.10 (0.94 - 1.28) | 1.04 (0.88 - 1.22) | 0.302 |
| Bi | reference | 1.19 (1.01 - 1.40) | 1.30 (1.10 - 1.55) | 1.30 (1.07 - 1.57) | **0.006** |
| Fe | reference | 0.99 (0.85 - 1.17) | 1.03 (0.88 - 1.21) | 1.05 (0.90 - 1.24) | 0.457 |
| I | reference | 0.99 (0.84 - 1.17) | 1.15 (0.98 - 1.35) | 1.26 (1.07 - 1.48) | **0.002** |

The multivariable associations were assessed using Cox regression, results are expressed as HR(95%CI) and *P* for trend. Regressions were adjusted for age, sex, BMI categories (normal, overweight, obese), education (low/medium/high), marital status (alone, in couple), smoking (never, former, current), alcohol consumption (none, 1-13, 14-27 and 28+ per week), hypertension (yes, no), diabetes (yes, no), urinary osmolarity (continuous), physical activity (never, once or twice per week), triglycerides, uric acid , C-reactive protein, 25-hydroxyvitamin D3 (all continuous), eGFR levels (continuous) and mean follow-up time (continuous). Q, quartile.

**Supplementary Table 4** Basic characteristics of included and excluded participants.

| **Variables** | **Included** | **Excluded** | ***P-value*** |
| --- | --- | --- | --- |
| Age, years | 51.9 ± 10.4 | 54.4 ± 11.2 | **<0.001** |
| Female sex, % | 2476(52.6) | 1068(52.6) | 1.000 |
| Education level, % |  |  | **<0.001** |
| Low | 2508(53.4) | 1266(62.7) |  |
| Middle | 1158(24.6) | 467(23.1) |  |
| High | 1033(22.0) | 287(14.2) |  |
| Marital status, % |  |  | 0.064 |
| Living alone | 1515(32.2) | 699(34.5) |  |
| Living in couple | 3187(67.8) | 1325(65.5) |  |
| Smoking status, % |  |  | 0.063 |
| Never | 1903(40.5) | 829(40.9) |  |
| Former | 1563(33.2) | 620(30.6) |  |
| Current | 1236(26.3) | 576(28.5) |  |
| Alcohol consumption, % |  |  | **<0.001** |
| None | 1262(26.8) | 653(32.2) |  |
| 1-13/week | 2655(56.5) | 998(49.2) |  |
| 14-27/week | 621(13.2) | 292(14.4) |  |
| 28+/week | 166(3.5) | 86(4.2) |  |
| BMI groups, % |  |  | **<0.001** |
| Normal | 2341(49.8) | 896(44.2) |  |
| Overweight | 1716(36.5) | 746(36.8) |  |
| Obese | 647(13.7) | 384(19.0) |  |
| Hypertension, % | 1574(33.5) | 926(45.8) | **<0.001** |
| Diabetes, % | 272(5.8) | 164(8.2) | **<0.001** |
| Lipids, mmol/L |  |  |  |
| Total cholesterol | 5.6 ± 1.0 | 5.6 ± 1.1 | 0.361 |
| LDL-C | 3.3 ± 0.9 | 3.4 ± 1.0 | 0.051 |
| HDL-C | 1.6 ± 0.4 | 1.6 ± 0.4 | **<0.001** |
| Triglycerides | 1.4 ± 1.1 | 1.5 ± 1.4 | **<0.001** |
| Serum creatinine, µmol/L | 79.8 ± 17.4 | 79.7 ± 28.2 | 0.896 |
| Urine creatinine, µmol/L | 152.8 ± 74.8 | 145.9 ± 77.2 | **<0.001** |
| Uric acid, mmol/L | 310.0 ± 84.5 | 317.9 ± 84.9 | **<0.001** |
| Urinary mOsm/H_2_O kg | 720.7 ± 212.9 | 699.9 ± 241.6 | **0.001** |
| C-reactive protein (mg/L) | 1.2[0.6-2.6] | 1.5[0.7-3.2] | **<0.001** |
| 25-OH vitamin D3(nmol/L) | 51.4 ± 24.1 | 48.0 ± 24.2 | **<0.001** |
| Physical activity |  |  | **<0.001** |
| Never | 1610(34.7) | 767(38.4) |  |
| Once a week | 486(10.5) | 165(8.2) |  |
| Twice a week | 2544(54.8) | 963(48.2) |  |
| Does not know | 0(0) | 104(5.2) |  |
| Renal Kidney function, % |  |  | **0.002** |
| Normal | 2415(51.3) | 1029(51.1) |  |
| IRF IkF | 2172(46.2) | 901(44.8) |  |
| CKD | 117(2.5) | 82(4.1) |  |
| eGFR, mL/min/1.73m2 | 89.7 ± 14.8 | 89.2 ± 16.3 | 0.221 |

Results are expressed as number of participants (column percentage) for categorical variables and as average ± standard deviation or median and [interquartile range] for continuous variables. Between-group comparisons were performed using chi-square for categorical variables and student’s t-test or Kruskal-Wallis test for continuous variables.

**Supplementary Table 5** Risk of rapid decline in kidney function at first follow-up.

| **Abbreviations** | **Rapid kidney function decline** | |
| --- | --- | --- |
|  | OR and (95% CI) | *P-value* |
| Li § | 1.00 (0.69 - 1.45) | 0.997 |
| Be | 1.02 (0.87 - 1.19) | 0.811 |
| Al | 1.03 (1.00 - 1.07) | 0.064 |
| V § | 1.13 (1.03 - 1.24) | **0.011** |
| Cr § | 1.03 (1.00 - 1.07) | 0.083 |
| Mn§ | 1.04 (0.99 - 1.08) | 0.132 |
| Co | 1.95 (0.88 - 4.30) | 0.100 |
| Ni | 0.98 (0.78 - 1.22) | 0.856 |
| Cu | 1.01 (0.97 - 1.07) | 0.570 |
| Zn § | 1.00 (0.96 - 1.04) | 0.929 |
| As § | 0.96 (0.76 - 1.22) | 0.747 |
| Se § | 1.32 (0.73 - 2.38) | 0.353 |
| Mo § | 1.94 (1.21 - 3.11) | **0.006** |
| Ag § | 1.56 (1.06 - 2.32) | **0.026** |
| Cd § | 1.02 (1.00 - 1.05) | 0.095 |
| Sn | 1.10 (0.83 - 1.45) | 0.523 |
| Sb | 0.43 (0.01 - 23.85) | 0.684 |
| Hg | 0.92 (0.77 - 1.10) | 0.369 |
| Pb | 1.06 (0.89 - 1.26) | 0.499 |
| Tl§ | 0.98 (0.89 - 1.07) | 0.586 |
| Bi | 1.00 (0.97 - 1.03) | 0.885 |
| Fe | 1.02 (0.98 - 1.05) | 0.402 |
| I § | 1.28 (1.07 - 1.53) | **0.008** |

The multivariable associations were assessed using logistic regression, results are expressed as OR and corresponding p-value. OR: odds ratio; § odds ratio for a 50-unit increase. Regressions were adjusted for age, sex, BMI categories (normal, overweight, obese), education (low/medium/high), marital status (alone, in couple), smoking (never, former, current), alcohol consumption (none, 1-13, 14-27 and 28+ per week), hypertension (yes, no), diabetes (yes, no), urinary osmolarity (continuous), physical activity (never, once or twice per week), triglycerides, uric acid , C-reactive protein, 25-hydroxyvitamin D3 (all continuous), eGFR levels (continuous) and mean follow-up time (continuous).

**Supplementary Table 6** Association between heavy metal/trace element levels and relative change in eGFR.

| **Metals/trace elements** | **Bivariate** | | **Multivariate** | |
| --- | --- | --- | --- | --- |
|  | Beta | *P-value* | Beta | *P-value* |
| Li | 0.019 | 0.192 | 0.022 | 0.17 |
| Be | 0.025 | 0.097 | 0.011 | 0.519 |
| Al | 0.054 | **<0.001** | 0.046 | **0.005** |
| V | 0.053 | **<0.001** | 0.044 | **0.007** |
| Cr | 0.034 | **0.021** | 0.011 | 0.477 |
| Mn | 0.05 | **0.001** | 0.043 | **0.031** |
| Co | 0.027 | 0.069 | 0.046 | **0.005** |
| Ni | 0.031 | **0.039** | 0.023 | 0.167 |
| Cu | 0.109 | **<0.001** | 0.093 | **<0.001** |
| Zn | 0.057 | **<0.001** | 0.009 | 0.574 |
| As | -0.026 | 0.081 | -0.016 | 0.307 |
| Se | 0.008 | 0.577 | 0.02 | 0.201 |
| Mo | 0.054 | **<0.001** | 0.082 | **<0.001** |
| Ag | 0.076 | **<0.001** | 0.084 | **<0.001** |
| Cd | 0.083 | **<0.001** | 0.038 | **0.042** |
| Sn | 0.038 | **0.01** | 0.035 | **0.031** |
| Sb | 0.019 | 0.212 | -0.004 | 0.821 |
| Hg | -0.03 | **0.045** | -0.012 | 0.439 |
| Pb | 0.036 | **0.016** | -0.001 | 0.958 |
| Tl | -0.006 | 0.692 | -0.001 | 0.993 |
| Bi | 0.031 | **0.04** | 0.01 | 0.598 |
| Fe | 0.039 | **0.008** | 0.005 | 0.765 |
| I | 0.064 | **<0.001** | 0.049 | **0.003** |

The relative change in eGFR is defined as the ratio between the absolute decrease and the initial values. The multivariable associations were assessed using linear regression, results are expressed as beta for the Metals/trace element and corresponding *p*-value. Regressions were adjusted for age, sex, BMI categories (normal, overweight, obese), education (low/medium/high), marital status (alone, in couple), smoking (never, former, current), alcohol consumption (none, 1-13, 14-27 and 28+ per week), hypertension (yes, no), diabetes (yes, no), urinary osmolarity (continuous), physical activity (never, once or twice per week), triglycerides, uric acid, C-reactive protein, 25-hydroxyvitamin D3 (all continuous), eGFR levels (continuous) and mean follow-up time (continuous).

**Supplementary material of Covariates**

We selected potential confounding factors based on the literature on the relationship between heavy metals and trace elements and kidney function. We selected age (years), sex (male/female), education (low/medium/high), marital status (living alone/living in couple), weekly alcohol consumption (units), smoking (Never/Former/Current), hypertension (yes/no), diabetes (yes/no), body mass index (BMI) (normal/overweight/obese), and urinary osmolarity (mOsm/H_2_O kg).

Education was categorized into high (university), middle (high school) and low (apprenticeship + mandatory). Marital status was defined as living alone (single, divorced, widowed) or living with a partner. Usual alcohol consumption during the week was self-reported and reported as number of units (glasses of wine, bottles or cans of beer, and shots of spirits) per week. Smoking was self-reported and categorized as never, former (irrespective of the time since quitting smoking) and current. Physical activities were categorized as never, once a week and twice a week.

Participants reported all medicines prescribed or bought over the counter. Medicines were coded according to the Anatomic, Therapeutic and Chemical (ATC) classification of the WHO. Lithium treatment was defined by codes N05AN* and D11AX04. Iodine containing drugs were defined by codes H03C*, D08AG* and H03AA*, where *=any code.

Body weight and height were measured with participants barefoot and in light indoor clothes. Body weight was measured in kilograms to the nearest 100 g using a Seca® scale (Hamburg, Germany). Height was measured to the nearest 5 mm using a Seca® (Hamburg, Germany) height gauge. Body mass index was calculated and categorized as normal (<25 kg/m^2^), overweight ≥25 and <30 kg/m^2^) and obese ≥30 kg/m^2^).

Blood pressure (BP) was measured thrice using an Omron® HEM-907 automated oscillometric sphygmomanometer after at least a 10-minute rest in a seated position, and the average of the last two measurements was used. Hypertension was defined by a SBP ≥140 mm Hg or a DBP ≥90 mm Hg or presence of antihypertensive drug treatment.

Glucose was assessed by glucose dehydrogenase. Diabetes mellitus (DM) was defined as fasting plasma glucose≥7.0 mmol/L and/or presence of oral hypoglycaemic or insulin treatment.
